# New Insights into How Trafficking Regulates T Cell Receptor Signaling

**DOI:** 10.3389/fcell.2016.00077

**Published:** 2016-07-26

**Authors:** Jieqiong Lou, Jérémie Rossy, Qiji Deng, Sophie V. Pageon, Katharina Gaus

**Affiliations:** ^1^EMBL Australia Node in Single Molecule Science, School of Medical Sciences, University of New South WalesSydney, NSW, Australia; ^2^ARC Centre of Excellence in Advanced Molecular Imaging, University of New South WalesSydney, NSW, Australia

**Keywords:** TCR signaling, exocytic trafficking, LAT, Rabs, SNAREs

## Abstract

There is emerging evidence that exocytosis plays an important role in regulating T cell receptor (TCR) signaling. The trafficking molecules involved in lytic granule (LG) secretion in cytotoxic T lymphocytes (CTL) have been well-studied due to the immune disorder known as familial hemophagocytic lymphohistiocytosis (FHLH). However, the knowledge of trafficking machineries regulating the exocytosis of receptors and signaling molecules remains quite limited. In this review, we summarize the reported trafficking molecules involved in the transport of the TCR and downstream signaling molecules to the cell surface. By combining this information with the known knowledge of LG exocytosis and general exocytic trafficking machinery, we attempt to draw a more complete picture of how the TCR signaling network and exocytic trafficking matrix are interconnected to facilitate T cell activation. This also highlights how membrane compartmentalization facilitates the spatiotemporal organization of cellular responses that are essential for immune functions.

## Introduction

**Figure 1:**
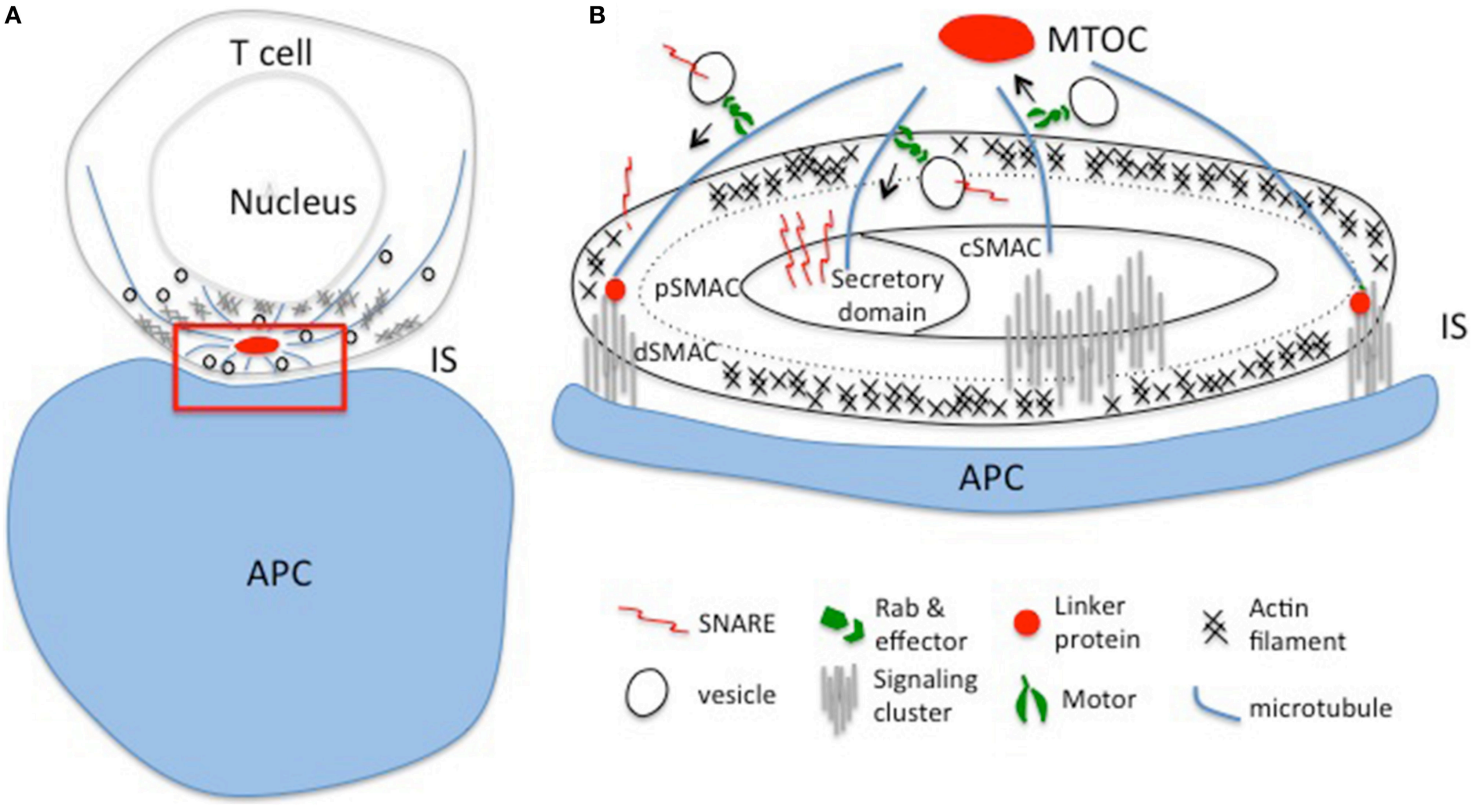
**Polarized exocytosis initiated by cytoskeleton reorganization upon TCR activation. (A)** T cell receptors encounter their cognate antigen on the APC leading to the formation of an immunological synapse and polarized intracellular trafficking established by the reorganization of actin and microtubule networks. Polarized intracellular trafficking is essential for directed release of effectors and local accumulation of signaling molecules at the immunological synapse. **(B)** Enlargement of the red region in panel **(A)**. Vesicles travel along actin and mainly microtubule networks beneath the synapse. Rabs and SNAREs, and linker proteins connect the cytoskeletal network with the plasma membrane to ensure precise spatio-temporal control of the delivery of effectors and signaling molecules to the different domains of the synapse: the secretory domain, the central (cSMAC), peripheral (pSMAC), and distal (dSMAC) central supramolecular activation clusters. IS, immunological synapse; APC, antigen presenting cell; MTOC, microtubule organizing center.

The key signaling molecules involved in the T Cell Receptor (TCR) signaling network have been well-characterized. T cell signaling is initiated upon TCR engagement by major histocompatibility complex (MHC) molecules bound to peptide antigens (pMHC). Upon TCR engagement, the TCR-associated CD3 dimers are phosphorylated by the kinase Lck on intracellular immunoreceptor tyrosine-based activation motif (ITAM) consensus sites, leading to the recruitment and activation of downstream signaling molecules, such as the adaptor protein Linker for Activated T cells (LAT). Subsequent activation responses including the secretion of lytic granules (LG) target infected or cancer cells for lysis. The formation of a structured interface between a T cell and an antigen-presenting cell (APC), termed the immunological synapse, is critical for the efficient delivery of effector molecules to the APC and intracellular signals in the T cell. The architecture of the immunological synapse was first described in 1998 as a “bull's eye pattern,” consisting of the central supramolecular activation cluster (cSMAC), where signaling molecules such as the TCR accumulated, surrounded by a ring of adhesion molecules known as the peripheral SMAC (pSMAC), with other molecules such as CD45 being excluded and localized in the distal SMAC (dSMAC, Figure 1; Monks et al., 1998; Grakoui et al., 1999; Freiberg et al., 2002).

Fluorescence microscopy has aided our understanding of the mechanisms underlying the coalescence and segregation of receptors and signaling molecules in the plasma membrane during synapse formation. Many studies focused on how the two-dimensional organization of proteins within the synapse contributes to T cell activation. However, as three-dimensional objects, cells can utilize an additional layer of regulatory mechanisms to control TCR signaling outcomes. Emerging evidence suggests that intracellular vesicular trafficking plays an important role in orchestrating TCR signaling. Engaged TCRs are internalized and targeted for degradation, but can also remain phosphorylated and signaling-competent (Luton et al., 1997; Coombs et al., 2002; Yudushkin and Vale, 2010; Benzing et al., 2013). Continuous delivery of the TCR into the immunological synapse is essential for sustained signaling and T cell activation (Grakoui et al., 1999; Lee et al., 2003; Soares et al., 2013b; Choudhuri et al., 2014; Martin-Cofreces et al., 2014). For example, although Lck activity is enhanced by TCR ligation (Stirnweiss et al., 2013), it is thought that the spatial organization and subcellular redistribution of Lck, in conjunction with antigen-binding induced conformational changes of the TCR-CD3 complex (Martinez-Martin et al., 2009; Swamy et al., 2016), control the extent of TCR-CD3 phosphorylation (Ehrlich et al., 2002; Thoulouze et al., 2006; Anton et al., 2008; Nika et al., 2010; Rossy et al., 2013). In the case of the adaptor protein LAT, the docking of sub-synaptic vesicles was observed in response to the initial wave of TCR signaling to sustain TCR signaling (Bonello et al., 2004; Billadeau, 2010; Purbhoo et al., 2010; Williamson et al., 2011; Larghi et al., 2013). In addition to the classical process of vesicles forming inside the cell, docking, and fusing at the immunological synapse, TCR-enriched microvesicles can also form and be released from the center of the IS to transmit signals to the APC (Choudhuri et al., 2014).

In T cells, there is a continuous flow of proteins and membranes along the endocytic and exocytic pathways. When a T cell encounters an APC, ligation of the TCR with cognate pMHC molecules leads to rapid cytoskeletal reorganization/polarization, which ensures the initiation of receptor and signaling protein endocytosis, and polarized secretion of LG and other vesicles (Dustin and Cooper, 2000; Angus and Griffiths, 2013). The internalization of the TCR-CD3 complex and downstream signaling proteins including LAT and SLP76 (SH2 domain containing leukocyte protein of 76 kDa) is relatively well-studied. Upon TCR activation, both engaged and bystander TCR can be internalized by clathrin independent, and/or dependent endocytosis (Monjas et al., 2004). Internalized engaged TCR-CD3 complexes are mainly degraded, while bystander TCR-CD3 complexes are predominately recycled back to the immunological synapse (Liu et al., 2000; Monjas et al., 2004; von Essen et al., 2004). SLP76 and LAT are first recruited to TCR and ZAP70 clusters in the plasma membrane and then segregate from these clusters during the transport of engaged TCR toward the central region of the immunological synapse within the membrane. This initiates LAT and SLP76 endocytosis (Barr et al., 2006). Internalized LAT were found to co-localize with transferrin positive vesicles, indicating a clathrin-mediated endocytic trafficking route, as well as cholera toxin B positive vesicles that do not co-localize with transferrin-positive vesicles in T cells and distinct SLP76-positive vesicles (Balagopalan et al., 2009). The E3 ubiquitin ligase Cbl and Cbl-b are known to play a key role in both TCR and LAT internalization (Naramura et al., 2002; Balagopalan et al., 2007). Noteworthy, no detectable LAT degradation seems to occur in response to TCR activation, indicating LAT is mainly recycled instead of being degraded after internalization, although LAT was ubiquitinated in response to TCR signaling (Balagopalan et al., 2007).

Vesicles containing cargo such as signaling molecules or effector molecules are transported along the microtubules or actin filament networks by molecular motors and delivered to the immunological synapse. The precise sorting and delivery of cargo are dependent on a subfamily of Ras GTPases, called Rab proteins (Fukuda, 2008; Wandinger-Ness and Zerial, 2014), and on membrane docking and fusion machinery proteins, known as soluble N-ethylmaleimide-sensitive-factor accessory-protein receptors (SNAREs; Das et al., 2004; Jahn and Scheller, 2006; Figure 1). Endosomal sorting complexes required for transport (ESCRT) components mediate the release into the extracellular space of vesicles that form at the immunological synapse (Choudhuri et al., 2014). In this review, we will summarize the known Rab and SNARE proteins involved in both the delivery of signaling molecules and the secretion of effectors at the immunological synapse in response to TCR activation. We will also summarize the known protein–protein interactions that may facilitate efficient and precise delivery of signaling molecule-containing vesicles in the activated TCR signaling network, using LAT-containing vesicles as an example.

## Exocytosis machinery of effector and signaling molecules

**Figure 2:**
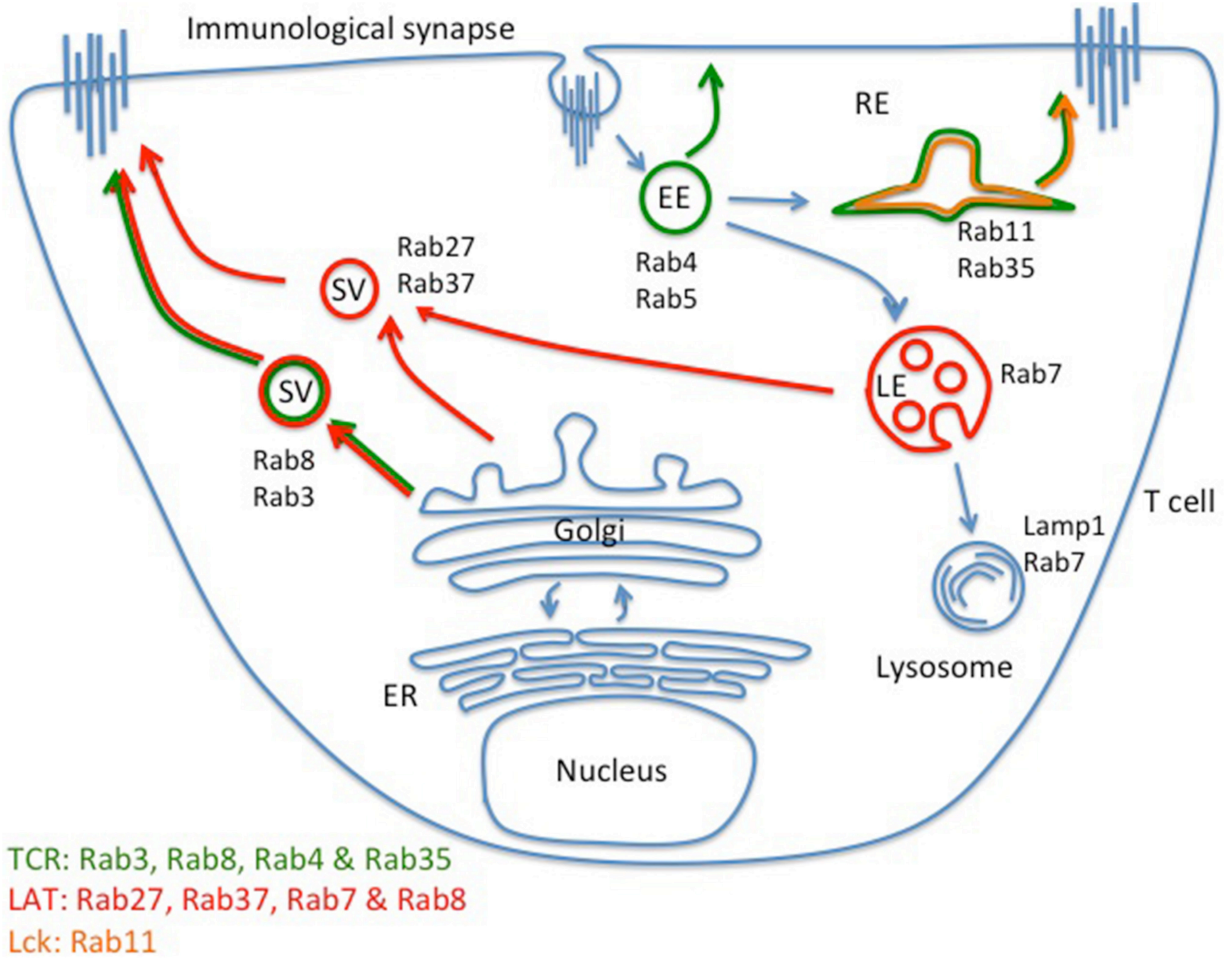
**Distinct intracellular compartments control TCR, LAT, and Lck exocytosis**. Regulated exocytosis of receptors and signaling molecules is initiated upon TCR activation. According to current understanding, exocytosis of the TCR-CD3 complex is mainly facilitated by newly formed Rab8- and Rab3-positive vesicles. Fast (Rab4-positive vesicles) and slow (Rab11-positive vesicles) recycling compartments can also contribute to the delivery of the TCR-CD3 complex to the immunological synapse. Lck co-localizes with Rab11-positive vesicles. LAT vesicles mainly contain the late endosome/lysosome markers of Rab27 and Rab37 but LAT also co-localizes with newly synthesized Rab8-positive vesicles. There appears to be little overlap between the TCR, LAT, and Lck exocytic trafficking pathways, indicating that distinct trafficking routes of signaling molecules may facilitate segregation before stimulation, and efficient delivery to and high number of encounters within the immunological synapse after stimulation. EE, early endosome; LE, late endosome; RE, recycling endosome; SV, secreting vesicle.

The TCR and downstream signaling proteins undergo constant internalization and exocytosis in resting T cells (Geisler, 2004). Upon TCR activation, polarized exocytosis is initiated by cytoskeletal rearrangements, which may be sufficient for receptors and signaling molecules to locally accumulate in the immunological synapse. So far, little is known about the molecular machinery involved in exocytosis of TCR and TCR signaling proteins, neither in resting nor in activated T cells. The monomeric G protein Rabs are the coordinators of intracellular membrane trafficking of TCR and downstream signaling proteins. Soares and colleagues have evaluated he role of 17 Rabs known to be involved in exocytic processes and examined TCR, Lck, and LAT exocytosis during TCR activation. They demonstrated that TCRζ can be delivered to the immunological synapse from fast recycling Rab4b compartments. A newly synthetized pool of TCR is also brought to the immunological synapse *via* Rab3d and Rab8b-positive compartments (Soares et al., 2013a). In addition, the TCR has been reported to co-localize with Rab35 and transferrin-positive compartments. It has been further shown that a Rab35-dominant negative mutation impairs TCR enrichment at the immunological synapse (Das et al., 2004; Patino-Lopez et al., 2008). Lck-containing vesicular compartments co-localized with the recycling endosome marker Rab11b (Soares et al., 2013a). Further, LAT vesicles co-localized with late endosome marker Rab7, newly synthesized protein pool Rab8 positive compartments, as well as Rab27a and Rab37, two Rab molecules known to regulate LG and cytokine secretion, respectively (Hong, 2005; Fischer et al., 2007; Purbhoo et al., 2010; Fukuda, 2013; Soares et al., 2013a; Figure 2). Hence, according to these studies, there is very little overlap between the identities of vesicles containing the TCR, Lck, and LAT, which suggest that distinct trafficking mechanisms exist for different signaling molecules. Hence, the intracellular reservoir of TCR, Lck, and LAT destined for transport to the plasma membrane and/or exocytosis are clearly distinct from each other, and range from slow and fast recycling compartments, to Golgi, and late endosome/lysosome (Figure 2). The contribution of this complex organization to T cell signaling and activation remains to be fully understood.

In general, GTP-bound active Rab proteins regulate many steps of intracellular membrane trafficking by recruiting different effectors to restricted membrane domains (Grosshans et al., 2006). Rab effectors are very diverse and can be divided into three groups according to the vesicle trafficking steps they are involved in, such as vesicle formation, trafficking along cytoskeletal networks, and vesicle fusion. Within the LG secretion pathway, the role of Rab27a and its effectors has been identified in relation to familial hemophagocytic lymphohistiocytosis (FHLH) and Griscelli syndrome type2 (GS; Fukuda, 2008, 2013; Krzewski and Cullinane, 2013). Granule exocytosis by cytotoxic T lymphocytes (CTLs) is defective in these patients, and this was found to be due to dysfunctional Rab27a (Menasche et al., 2000; Haddad et al., 2001). Rab27a effectors are synaptotagmin-like proteins (Slp) and typically contain a Slp homology domain (SHD, Rab27a-binding domain) and two C2 domains (for binding to phospholipids and potentially calcium). Slp3-a forms a complex with Rab27a and the motor protein kinesin-1 and was demonstrated to mediate the terminal transport of LG to the immune synapse (Kurowska et al., 2012). There is evidence that Slp2-a is also involved in docking of LG in CTLs by binding to Rab27a and the plasma membrane (Menasche et al., 2008). Mutations of another Rab27a effector (Holt et al., 2008), Munc13-4, caused immunodeficiency in patients with type 3 FHLH. Munc13-4 plays a role in LG maturation and also drives the SNARE assembly process and is critical for the priming/fusion step of LG exocytosis (Feldmann et al., 2003). In addition, Munc13-4 has been also demonstrated to mediate fusion of Rab11-positive recycling vesicles with Rab27-positive late vesicles, constituting a pool of vesicles destined for regulated exocytosis (Menager et al., 2007). Munc18-2 promotes SNARE complex assembly (Hackmann et al., 2013; Spessott et al., 2015) and is not a direct effector but binds to another Rab27a effector, Slp-4a (Fischer et al., 2007; Jenkins and Griffiths, 2010; Hackmann et al., 2013; Krzewski and Cullinane, 2013).

Until now, limited information has been obtained regarding the role of Rab effectors in the regulation of exocytosis of TCR and signaling molecules. Table 1 summarizes the known effectors that are expressed in T cells for Rabs involved in exocytosis of TCR and signaling molecules (Grosshans et al., 2006; Fukuda, 2013; Wandinger-Ness and Zerial, 2014). Through Rab effectors, the Rab network is connected to the SNARE system, linking the transportation of exocytic vesicles in the cytosol to their docking and fusion with the plasma membrane. Prior to membrane fusion, SNAREs on opposing membranes are able to form four-helix bundles that lead to a tight connection of vesicular and target membranes. SNARE complexes can be divided into two groups, vesicle SNAREs (vSNAREs), and target membrane SNAREs (tSNAREs). SNAREs can also be divided into either Q- (Qa, Qb, Qc, or Qbc, mainly tSNAREs) or R-SNAREs (mainly vSNAREs) based on their structure. In general, the four helix-bundle of a functional SNARE complex must consist of one Qa-SNARE, one Qb-SNARE, one Qc-SNARE, and one R-SNARE. In some cases, the two helices of the Qb- and the Qc-SNARE can be provided by one protein (Jahn and Scheller, 2006). In addition to the core SNARE complex, the calcium sensors synaptotagmins, and synaptotagmin-like proteins facilitate the assembly of the SNARE complex and trigger the final membrane fusion step.

**Table 1:** **Effectors for Rabs involved in the exocytosis of TCR and TCR signaling molecules**.

**Signaling molecule**	**Rabs**	**Effectors**	**Effector expression in T cell**	**Proposed functions**
TCR	Rab3d	Regulating synaptic membrane exocytosis 1/2(RIMS1/2)	Low/medium	Regulates synaptic vesicle exocytosis, regulates voltage-gated calcium channels, scaffolding protein (Millar et al., 2002; Gandini et al., 2011)
		Rabphilin 3A like (without C2 domain; RPH3AL)	Low	A direct regulatory role in calcium ion-dependent exocytosis (Li et al., 1994; Millar et al., 2002; Grosshans et al., 2006)
	Rab4b	Rabaptin, Rab GTPase-binding effector protein 1 (RabEP1)	High	Acts as a linker between adaptin and Rab4 and Rab5, involved in endocytic membrane fusion (Vitale et al., 1998)
		RUN and FYVE domain containing 1 (RUFY1)	Medium	Binds to phospholipid vesicles and participates in early endosomal trafficking (Cormont et al., 2001; Fouraux et al., 2004)
		RAB11 family-interacting protein 1 (RAB11FIP1)	Medium	Regulates Rab GTPases (Lindsay et al., 2002)
		CD2-associated protein (CD2AP)	Medium	Regulates actin cytoskeleton (Kirsch et al., 1999; Cormont et al., 2003)
		GRIP1 associated protein 1 (GRIPSP1)	Medium	Interacting with endosomal SNARE syntaxin 13 (Hoogenraad et al., 2010)
	Rab8b	Otoferlin (OTOF)	Low	Calcium sensor, regulates vesicle membrane fusion in calcium-dependent manner (Roux et al., 2006)
		Synaptotagmin-like 1 (SYTL1)/SLP1/exophilin-7	Medium	Binds to PI3,4,5P3 (Hattula et al., 2006)
		Optineurin/RAB11 family-interacting protein 2 (OPTN/RAB11FIP2)	Medium	Interacts with myosin VI (Ying and Yue, 2012)
		Mitogen-activated protein kinase kinase kinase kinase 2 (MAP4K2)	Medium	Serine/threonine protein kinase (Katz et al., 1994; Ren et al., 1996)
		MICAL (microtubule associated monooxygenase, calponin, and LIM domain containing)-like 1 (MICALL1)	Medium	Linking EHD1 and Rab8 on recycling endosomal membrane tubules (Sharma et al., 2009)
		MICAL (microtubule associated monooxygenase, calponin, and LIM domain containing)-like 2 (MICALL2)/junctional Rab13-binding protein (JRAB)	Low	Regulates the endocytic recycling of occludins, claudins and E-cadherin to the plasma membrane, may regulate actin cytoskeleton (Yamamura et al., 2008)
		Oculocerebrorenal syndrome of Lowe (OCRL)	Mixed reports of low and high expression	Phosphatase enzyme involved in actin polymerization and may function in lysosomal membrane trafficking (Hagemann et al., 2012; Luo et al., 2012)
		Myosin 5B (MYO5B)	Low	Motor protein, travels toward the plus end of actin filaments (Khandelwal et al., 2013)
	Rab35	Oculocerebrorenal syndrome of Lowe OCRL	Mixed reports of low and high expression	Phosphatase enzyme involved in actin polymerization and may function in lysosomal membrane trafficking (Dambournet et al., 2011)
		Fascin actin-bundling protein 1 (FSCN1)	Medium	Actin crosslinking protein (Zhang et al., 2009)
		Run and SH3 domain containing 1 (RUSC1/NESCA)	Low	RUN and SH3 domain containing protein (Fukuda et al., 2011; Chaineau et al., 2013)
		Microtubule associated monooxygenase, Calponin and LIM domain containing 1 (MICAL1)	Medium/High	Disassemble actin filament (Chaineau et al., 2013)
		MICAL like protein 1 (MICAL-L1)	Medium/High	Interacting with EHD1 (Rahajeng et al., 2012)
		ArfGAP with coiled-coil, Ankyrin repeat and PH domains 2 (ACAP2)	Medium	Arf GAP (Kobayashi and Fukuda, 2012)
Lck	Rab11b	Optineurin/RAB11 family-interacting protein 2 (OPTN/RAB11FIP2)	Medium	Interacts with myosin VI (Wandinger-Ness and Zerial, 2014)
		Phosphatidylinositol 4-kinase beta (PI4KB)	High	Regulates the trafficking from Golgi to plasma membrane (de Graaf et al., 2004)
		TBC1 domain family membrane 14 (TBC1D14)	Low	Regulates autophagosome formation (Longatti et al., 2012)
		WD repeat domain 44/Rabphilin-11/rab11-binding protein (WDR44)	High	Plays a role in endosome recycling (Wandinger-Ness and Zerial, 2014; Vetter et al., 2015)
		Zinc finger FYVE domain-containing 27 (ZFYVE27)	Medium	Functions as an upstream inhibitor of Rab11 (Shirane and Nakayama, 2006)
		Exocyst complex component 6 (EXOC6)/Sec15	Medium	Essential for vesicular traffic from Golgi apparatus to the cell surface (Zhang et al., 2004; Wu et al., 2005)
		Myosin 5B (MYO5B)	Low	Motor protein, travels toward the plus end of actin filaments (Roland et al., 2011)
LAT	Rab27a	Synaptotagmin-like 2 (SYTL2)/SLP2-a/exophilin-4	High	Calcium sensor, binds to PS, PI(4,5)P2 (Galvez-Santisteban et al., 2012)
		Synaptotagmin-like 1 (SYTL1)/SLP1/exophilin-7	Medium	Calcium sensor, binds to PI(3,4,5)P3 (Brzezinska et al., 2008)
		Synaptotagmin-like 3 (SYTL3)/SLP3-a/exophilin-6	Low	Forms Rab27a/kinesin-1/SYTL3 complex (Gibbs et al., 2004; Fukuda, 2013)
		Unc-13 homolog D (Unc13d)/Munc13-4	High	Controls the priming/fusion step of LG exocytosis (Feldmann et al., 2003; Brzezinska et al., 2008)
		Myosin 5B (MYO5B)	Low	Travels toward the plus end of actin filaments (Gibbs et al., 2004; Fukuda, 2013)
		Rabphilin 3A like (without C2 domain; RPH3AL)	Low	A direct regulatory role in calcium ion-dependent exocytosis (Izumi, 2007; Fukuda, 2013)
		Melanophilin (MLPH)	Medium	Forms a ternary complex with Rab27a and myosin Va (Strom et al., 2002)
		Coronin, actin-binding protein, 1C (CORO1C)	Low	Binds to GDP-bound form of Rab27a, regulates endocytosis of secretory membrane (Kimura et al., 2008)
	Rab37	Regulating synaptic membrane exocytosis 1 (RIMS1)	Low	Regulates synaptic vesicle exocytosis, regulates voltage-gated calcium channels, scaffolding protein (Wandinger-Ness and Zerial, 2014)

Pattu and colleagues have examined the expression of 28 SNAREs and their co-localization with LGs and TCR-CD3 complex in synapses in primary human CD8+ T cells. They found that Stx16 Vtib and Stx8 had the highest co-localization with LGs while TCR-CD3 co-localized with Stx3, Stx4, Stx7, Stx13, Vtib, Stx6, Stx8, VAMP3, and VAMP4 (Pattu et al., 2012). In other reports, vSNARE VAMP2, VAMP8, tSNARE Stx7, Stx 11 Vti1b were found to be involved in LG secretion (Dressel et al., 2010; Pattu et al., 2011; Qu et al., 2011; Halimani et al., 2014; Marshall et al., 2015). For TCR exocytosis, vSNARE, VAMP2, VAMP3, VAMP7 as well as tSNARE Vti1B have been shown to play a role (Das et al., 2004; Pattu et al., 2011; Qu et al., 2011; Matti et al., 2013; Soares et al., 2013a; Finetti et al., 2015b). In Jurkat cells, tSNARE SNAP23 and Stx4 accumulated at the immunological synapse indicating that those SNAREs may also play a role in regulated exocytosis upon TCR activation (Das et al., 2004). Exocytosis of Lat vesicles in response to TCR activation relies on VAMP7 and synaptotagmin 7 (Syt7). Although vesicle fusion is the classical role of Syt7, no evidence has emerged to date that VAMP7- and Syt7-positive LAT vesicles fuse with the plasma membrane but it remains an open question whether LAT vesicles in activated T cells dock at or fuse with the plasma membrane (Williamson et al., 2011; Larghi et al., 2013; Soares et al., 2013a). Further work is required to draw a more complete picture of how SNARE complexes regulate the secretion of LG and the exocytosis of receptors and signaling molecules. The ultimate goal is to understand how such a great diversity and redundancy in the tethering-fusion apparatus during T cell activation regulate the fusion of TCR, Lat, or Lck containing vesicles to specific areas of the immunological synapse.

## Additional trafficking mechanisms for TCR signaling molecules

In addition to the classical machinery mediating trafficking—facilitated by Rab proteins—and fusion to the plasma membrane—facilitated by SNARES—other proteins have also been demonstrated to regulate the targeting of TCR, Lck, and LAT to the immune synapse. Sorting nexin 17 (SNX17) and SNX27 are implicated in recycling TCR toward the immunological synapse (Rincon et al., 2011; Osborne et al., 2015). Intraflagellar transport (IFT) particles generally mediate the assembly of cilia, but in T cells, IFT20 was found to co-localize with the microtubule organizing center (MTOC), and Golgi and post-Golgi compartments. Knocking down IFT20 resulted in the TCR-CD3 complex accumulating in Rab5 endosomes and failure to be properly recycled toward the immunological synapse (Finetti et al., 2009, 2015a; Finetti and Baldari, 2013). Recently, Vivar and colleagues demonstrated that IFT20 was also required for the delivery of the intracellular pool of LAT to the immunological synapse in primary murine CD4+ T cell (Vivar et al., 2016). The polarized recycling of TCR signaling proteins appears to be highly regulated. Indeed, Lck delivery to the immunological synapse relies on Unc119, which controls the transportation of Lck-positive endosomal compartments by regulating Rab11 activation, and orchestrating the recruitment of the actin-based motor protein, myosin 5B (Gorska et al., 2009). In addition, the membrane protein MAL, which is redistributed to the cSMAC upon T cell activation, contributes to target the delivery of Lck and LAT to the center of the immunological synapse (Anton et al., 2008, 2011). Microtubule plus end binding protein EB1 may contribute in directing LAT vesicles to the TCR-ZAP70 signaling complex. Indeed, in activated cells, knocking down EB1 impairs TCR clustering at the plasma membrane and the sustained activation of LAT and PLCγ1. But more importantly, the absence of EB1 prevents the encounters between LAT vesicles and CD3 vesicles at the immunological synapse (Martin-Cofreces et al., 2012).

Together, these studies show that several regulators are involved in fine-tuning how and when vesicles from the recycling pathway are redirected to bring components of the TCR signaling pathway to the immunological synapse. This suggests that targeted recycling has a critical role in T cell activation, which probably goes beyond the mere shuttling of membrane proteins to the plasma membrane, and might contribute to the spatiotemporal regulation of TCR signaling.

## Trafficking vs. signaling—the example of LAT-positive vesicles

LAT is an essential adaptor protein that is recruited to phosphorylated TCR-CD3 complexes at the cell surface. The kinase ZAP70 is responsible for phosphorylating tyrosine residues in cytoplasmic tail of LAT. Due to the nine tyrosine residues, LAT acts as a scaffolding protein for downstream signaling molecules including SLP-76, PLC-γ1, Grb2, Gads, Sos1, and so on. Recently the interactome of LAT was mapped out, which revealed 112 unique interactions in the ZAP70-LAT-SLP-76 signaling axis (Malissen et al., 2014). Previous imaging and biochemistry studies identified two pools of LAT, a cytoplasmic vesicular pool, and a plasma membrane pool that forms nano-clusters. To date, the functional differences between these two pools of LAT is not clear but the notion was put forward that LAT at the plasma membrane is involved in initial TCR signaling while vesicular LAT is required for signal amplification (Bonello et al., 2004; Purbhoo et al., 2010; Williamson et al., 2011; Balagopalan et al., 2013; Larghi et al., 2013; Soares et al., 2013a).

With respect to LAT vesicles, calcium fluxes in the context of Lck-mediated TCR signaling have emerged as one of the key factors in regulating the trafficking of these vesicles. Calcium influx initiated by the first wave of TCR signaling triggers the sub-synaptic LAT vesicle exocytosis which further propagates TCR signaling (Soares et al., 2013a). TCR activation initiates Lck spatial reorganization that conditions subsequent LAT vesicle delivery. Interfering with Lck translocation by knocking down MAL protein impairs LAT vesicle exocytosis. However it can be rescued by artificially creating calcium influx (Soares et al., 2013a). Reducing LAT exocytosis, by interfering with Lck exocytosis, or silencing calcium sensor Syt7 or vesicle SNARE VAMP7 decreased LAT phosphorylation and IL2 production in general (Williamson et al., 2011; Larghi et al., 2013; Soares et al., 2013a). Single molecule localization microscopy (SMLM) revealed that LAT and pLAT clusters number as well as cluster size decreased by blocking LAT vesicle exocytosis during TCR activation (Larghi et al., 2013). Moreover, the presence of interacting signaling nano-territories between LAT and SLP76 was also impaired by blocking LAT exocytosis (Soares et al., 2013a). Interestingly, the long, tubular-shaped LAT clusters almost disappeared when LAT exocytosis was blocked (Soares et al., 2013a). These long tubular LAT vesicles travel toward the immunological synapse during TCR activation (Bonello et al., 2004; Billadeau, 2010), supporting the idea that LAT vesicles do not fuse with the immunological synapse. Larghi et al. expressed LAT molecule that presented a HA-TEV-tag on the extracellular site. By cleaving the HA tag with TEV protease in plasma membrane LAT population before T cell activation, despite the enrichment of HA-TEV-LAT vesicles at the sub-synaptic membrane after TCR stimulation, LAT was not recognized with antibodies to HA in non-permeabilized cells, indicating that HA-TEV-LAT vesicles had not fused with the plasma membrane (Larghi et al., 2013). Since LAT vesicles appear to carry phosphorylated LAT in activated T cells (Williamson et al., 2011), it is possible that vesicular LAT is phosphorylated in *trans* by the TCR-ZAP70 complex in the plasma membrane. Given that LAT vesicles appear to dock at the plasma membrane for only ~1 min (Purbhoo et al., 2010; Williamson et al., 2011), many LAT molecules could be rapidly phosphorylated in that manner, leading to signal amplification and spreading throughout the cell.

## Conclusion

Extensive research has focused on understanding the TCR signaling network and signal-regulating mechanisms during T cell activation due to the essential roles of T cells in the adaptive immune system. Surprisingly, current knowledge of the trafficking machineries involved in the intracellular movement of TCR and signaling molecules remains very limited. In this review, we attempted to summarize the known trafficking machinery involved in the exocytosis of TCR, downstream signaling molecules and effectors during TCR activation. By putting this information into context, we tried to draw a more complete picture of trafficking networks involved in the regulation of TCR signaling.

It emerges that key signaling molecules involved in early TCR signaling reside in distinct vesicle subpopulations that contain non-overlapping SNARE molecules (Das et al., 2004; Pattu et al., 2012; Larghi et al., 2013; Matti et al., 2013; Finetti et al., 2015b). Despite their differences, it seems that fusion with the plasma membrane might not be the final destination of some populations of vesicles, raising the question of how trafficking *per se* can contribute to regulate T cell signaling. Rather than distributing signaling molecules across the cell, and regulating signaling by changing the local concentration of available signaling molecules, vesicles themselves may be signaling entities. This is illustrated in the emerging concept of LAT vesicles that do not appear to fuse with the plasma membrane but are nevertheless phosphorylated upon TCR stimulation (Williamson et al., 2011; Larghi et al., 2013). Such vesicles resemble signaling endosomes (Benzing et al., 2013) but contain the trafficking machinery for exocytosis (Larghi et al., 2013; Soares et al., 2013a). Thus, LAT vesicles blur the lines between exocytosis, endocytosis, and signaling. Quantitative imaging may reveal whether LAT vesicles indeed amplify the initial TCR signals in the coming years. This would constitute a new perspective, as the trafficking machinery is not solely used to deliver molecules to and from the plasma membrane but to directly facilitate the signaling process in a highly controlled manner.

It is tempting to speculate that the different trafficking machinery is required to segregate Lck and LAT into different vesicles from those that contain TCR and the CD3 complex. Such segregation may be necessary to achieve the distinct spatial organization of the immunological synapse. Emerging imaging techniques such as the lattice light-sheet microscope (Chen et al., 2014) may soon reveal how vesicle trafficking is functionally linked to synapse organization. Segregation into different membrane compartments could both prevent signaling in resting T cells and facilitate sustained signaling in activated T cells. Already the actin cytoskeleton at the immunological synapse has been identified as a gatekeeper for the secretion of LG (Ritter et al., 2015) and new rapid, super-resolution imaging will undoubtedly bring further insights into the interconnectedness between the structural organization of the synapse, signaling activities, compartmentalization of signaling components, and vesicle movement.

## Author contributions

JL and QD wrote the manuscript, KG, JR, and SP provided critical reviewing and editing.

## Funding

We acknowledge funding from the Australia Research Council (CE14010011 to KG and DE140101626 to JR) and National Health and Medical Research Council of Australia (APP1037320 and APP1059278 to KG and APP1102730 to JR).

### Conflict of interest statement

The authors declare that the research was conducted in the absence of any commercial or financial relationships that could be construed as a potential conflict of interest.
